# New prognostic markers revealed by RNA-Seq transcriptome analysis after *MYC* silencing in a metastatic gastric cancer cell line

**DOI:** 10.18632/oncotarget.27208

**Published:** 2019-10-08

**Authors:** Luana de O. Lopes, Jersey H. Maués, Hygor Ferreira-Fernandes, France K. Yoshioka, Severino C. Sousa Júnior, Alcemir R. Santos, Helem F. Ribeiro, Juan A. Rey, Paulo C. Soares, Rommel R. Burbano, Giovanny R. Pinto

**Affiliations:** ^1^ Genetics and Molecular Biology Laboratory, Federal University of Piauí, Parnaíba, Brazil; ^2^ Laboratory of Molecular Biology, Ophir Loyola Hospital, Belém, Brazil; ^3^ Course of Medicine, Federal University of Piauí, Parnaíba, Brazil; ^4^ Computer Science Department, State University of Piauí, Piripiri, Brazil; ^5^ Institute of Biological Sciences, Federal University of Pará, Belém, Brazil; ^6^ Molecular Oncogenetics Laboratory, Hospital Universitario La Paz, Madrid, Spain

**Keywords:** stomach neoplasms, MYC oncogene, transcriptome, gene expression profiling, prognosis

## Abstract

*MYC* overexpression is considered a driver event in gastric cancer (GC), and is frequently correlated with poor prognosis and metastasis. In this study, we evaluated the prognostic value of genes upregulated by MYC in patients with GC. Metastatic GC cells (AGP01) characterized by *MYC* amplification, were transfected with siRNAs targeting *MYC*. RNA-seq was performed in silenced and non-silenced AGP01 cells. Among the differentially expressed genes, *CIAPIN1*, *MTA2*, and *UXT* were validated using qRT-PCR, western blot, and immunohistochemistry in gastric tissues of 213 patients with GC; and their expressions were correlated with clinicopathological and survival data. High mRNA and protein levels of *CIAPIN1*, *MTA2*, and *UXT* were strongly associated with advanced GC stages (*P* < 0.0001). However, only *CIAPIN1* and *UXT* gene expressions were able to predict distant metastases in patients with early-stage GC (*P* < 0.0001), with high sensitivity (> 92%) and specificity (> 90%). Overall survival rate of patients with overexpressed *CIAPIN1* or *UXT* was significantly lower (*P* < 0.0001). In conclusion, *CIAPIN1* and *UXT* may serve as potential molecular markers for GC prognosis.

## INTRODUCTION

Gastric cancer (GC) is the fifth most frequently diagnosed cancer worldwide [1]. Despite advances in the understanding of molecular mechanisms involved in gastric carcinogenesis [2], the early detection and prognostic outcomes of this disease, which will eventually define therapeutic success, still depend on histopathological methods. In the absence of specific symptoms suggestive of early-stage GC, and a lack of effective diagnostic imaging methods, most patients are diagnosed at advanced tumor stages, wherein, despite surgical resection, chemotherapy treatments are of only palliative nature [3]. Only 20–25% of patients with advanced GC survive 5 years after diagnosis [4], making GC one of the leading causes of cancer-related deaths worldwide (783,000 deaths estimated in 2018) [1].

Some recent studies have identified typical molecular alterations, such as *MYC* gene (8q24) amplification, as drivers of genetic profiles characterizing advanced stages of gastric tumors, with poor prognostic features [5–7]. MYC protein influences approximately 15% of the genes in the human genome through its interaction with enhancer box sequences (E-box), and via the recruitment of histone acetyltransferases. Deregulation of *MYC* gene expression promotes genomic instability, and high levels of MYC protein have been shown to create a mutagenic environment by increasing the levels of reactive oxygen species [8].

Cell lines play an important role in the study of molecular patterns associated with carcinogenesis and metastasis. A cancer cell line, designated as AGP01, was established by our research group from ascitic fluid cells of a patient with metastatic gastric adenocarcinoma. AGP01 cells are characterized by clonal chromosomal abnormalities, such as trisomy 8, resulting in the amplification of *MYC* gene [9]. Given the important role of MYC in GC prognosis, analysis of MYC-regulated genes may provide valuable biomarkers for GC risk stratification, which can help in the treatment choice. Therefore, the objective of this study was to evaluate the prognostic and predictive values of genes upregulated by MYC overexpression, selected from high-throughput RNA sequencing (RNA-seq) data, in a metastatic gastric adenocarcinoma cell line (AGP01), before and after siRNA-mediated *MYC*-silencing.

## RESULTS

### *MYC* silencing in AGP01 cell line and RNA-seq

**Figure 1:**
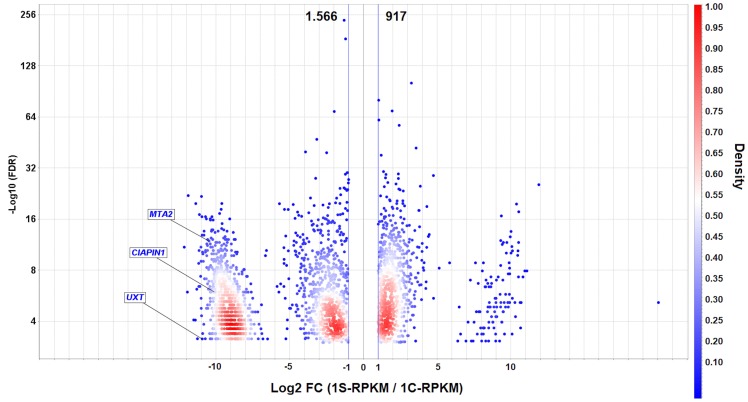
Volcano plot of differentially expressed genes (DEGs) in AGP01 cell line upon *MYC* silencing. A direct comparison between *MYC*-silenced and non-silenced cells is shown. -Log10 (FDR) ≤ 0.05 (Y-axis) and the cut-off point |Log2(FC)| ≥ 1 (X-axis) indicate the downregulated (left side) and upregulated (right side) DEGs. The *UXT*, *CIAPIN1*, and *MTA2* genes are highlighted as the significantly downregulated genes. The density is calculated to visualize the gene overlap. RPKM: Reads per kilo base per million mapped reads.

A total of 11 and 13 million RNA-seq reads generated respectively from *MYC*-silenced and non-silenced AGP01 cells, revealed 2,483 differentially expressed genes (DEGs), of which 917 were upregulated, and 1,566 were downregulated due to *MYC* silencing. The downregulated DEGs represented the genes, whose overexpression was influenced, directly or indirectly, by the high levels of MYC in AGP01 cell line. Since *MYC* amplification is a common phenomenon in patients with GC, it is reasonable to infer that those genes may also be overexpressed in the tumor tissues of patients. Thus, we randomly selected 3 genes from 150 most downregulated DEGs (Supplementary Table 1) to assess their prognostic and predictive value in GC clinical samples. The selected genes were as follows: *CIAPIN1* (cytokine induced apoptosis inhibitor 1), *MTA2* (metastasis associated 1 family member 2), and *UXT* (ubiquitously expressed prefoldin like chaperone) (Figure 1).

### Clinicopathological features and *CIAPIN1*, *MTA2*, and *UXT* expression

**Figure 2:**
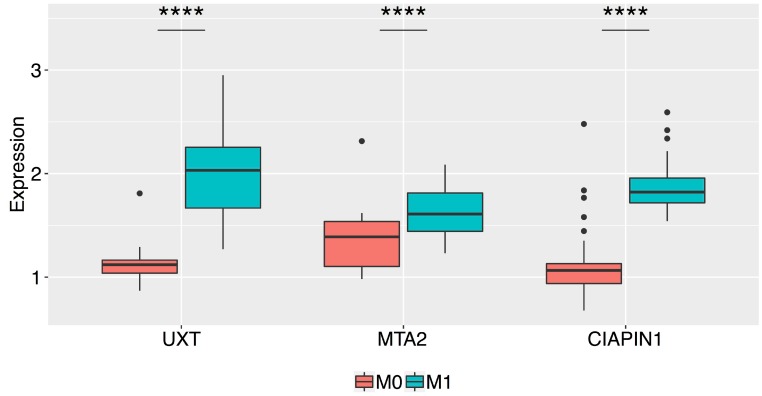
Box plots of the normalized relative expression of the UXT, MTA2, and CIAPIN1 proteins in the gastric tumor tissue of patients without metastasis (M0) and with metastasis (M1) (^****^ *P* < 0.0001). The boxes are drawn from the 75th percentile to the 25th percentile. The horizontal line inside the box represents median values. The vertical lines above and below the box delineate the maximum and minimum values, and the dots indicate outliers.

The relative mRNA expressions of *CIAPIN1*, *MTA2*, and *UXT* genes in the tumor tissues of patients with various clinicopathological features are shown in Table 1. The expression of all the 3 genes was significantly higher in the following scenarios (compared with paired normal gastric tissues): serosal invasion-positive (T3/T4) (*CIAPIN1*, 1.888 ± 0.547; *MTA2*, 2.034 ± 0.375; *UXT*, 1.784 ± 0.656, ^****^ *P* < 0.0001), positive lymph node metastasis (N1) (*CIAPIN1*, 1.875 ± 0.592; *MTA2*, 1.871 ± 0.462; *UXT*, 1.656 ± 0.552, ^****^ *P* < 0.0001), and positive distant metastasis (M1) (*CIAPIN1*: 2.292 ± 0.452; *MTA2*, 2.016 ± 0.462; *UXT*, 2.010 ± 0.482, ^****^ *P* < 0.0001). *MTA2* expression was higher in patients aged ≥61 years (1.880 ± 0.433, ^*^ *P* = 0.024), and in patients with intestinal GC (1.898 ± 0.451, ^**^ *P* = 0.005). We also observed an excessively high expression of all the three selected genes in M1 patients as compared to that in patients without metastasis (M0) (an increase of 70.5% for *CIAPIN1*, 25% for *MTA2*, and 67.8% for *UXT*; ^****^ *P* < 0.0001), and the data was corroborated by protein expression analysis (Figure 2).

**Table 1:** Relationship between *UXT*, *MTA2*, and *CIAPIN1* mRNA expression and clinicopathological features

Variables	*n*, 213 (%)	*UXT* expression^*^			*MTA2* expression^*^			*CIAPIN1* expression^*^		
		Mean ± SD	*F* value	*P* value	Mean ± SD	*F* value	*P* value	Mean ± SD	*F* value	*P* value
**Gender**										
Male	133 (62.4%)	1.566 ± 0.559	1.185	0.277	1.811 ± 0.450	0.002	0.957	1.788 ± 0.592	0.542	0.462
Female	80 (37.6%)	1.651 ± 0.528			1.814 ± 0.509			1.850 ± 0.592		
**Age (years)**										
≥61	113 (53.1%)	1.600 ± 0.524	0.004	0.946	1.880 ± 0.433	5.135	0.024^*^	1.858 ± 0.618	1.496	0.222
<61	100 (46.9%)	1.595 ± 0.576			1.735 ± 0.503			1.759 ± 0.558		
**Tumor location**										
Cardia	64 (30.0%)	1.615 ± 0.547	0.087	0.767	1.789 ± 0.497	0.216	0.642	1.840 ± 0.624	0.209	0.647
Non-cardia	149 (70.0%)	1.591 ± 0.550			1.822 ± 0.462			1.799 ± 0.578		
**Histological type**										
Diffuse	103 (48.4%)	1.634 ± 0.601	0.846	0.358	1.720 ± 0.479	7.714	0.005^**^	1.800 ± 0.606	0.066	0.796
Intestinal	110 (51.6%)	1.565 ± 0.493			1.898 ± 0.451			1.821 ± 0.580		
**Serosal invasion (T)**										
T1/T2	68 (31.9%)	1.511 ± 0.467	12.037	<0.0001^****^	1.338 ± 0.262	190.215	<0.0001^****^	1.647 ± 0.650	7.975	<0.0001^****^
T3/T4	145 (68.1%)	1.784 ± 0.656			2.034 ± 0.375			1.888 ± 0.547		
**Lymph node metastasis (N)**										
Negative	23 (10.8%)	1.123 ± 0.112	21.238	<0.0001^****^	1.326 ± 0.187	31.180	<0.0001^****^	1.282 ± 0.179	22.780	<0.0001^****^
Positive	190 (89.2%)	1.656 ± 0.552			1.871 ± 0.462			1.875 ± 0.592		
**Distant metastasis (M)**										
M0	108 (50.7%)	1.198 ± 0.203	258.523	<0.0001^****^	1.613 ± 0.392	47.119	<0.0001^****^	1.344 ± 0.216	383.538	<0.0001^****^
M1	105 (49.3%)	2.010 ± 0.482			2.016 ± 0.462			2.292 ± 0.452		
**TNM stage**										
I + II	42 (19.7%)	1.266 ± 0,210	21.026	<0.0001^****^	1.306 ± 0.220	83.330	<0.0001^****^	1.266 ± 0.220	55.912	<0.0001^****^
III + IV	171 (80.3%)	1.680 ± 0.574			1.936 ± 0.433			1.945 ± 0.577		
***H. pylori* infection **										
Negative	23(10.8%)	1.639 ± 0.623	0.140	0.708	1.790 ± 0.487	0.0576	0.810	1.710 ± 0.526	0.752	0.386
Positive	190 (89.2%)	1.593 ± 0.540			1.815 ± 0.471			1.824 ± 0.599		
***CagA**^a^*										
Negative	73 (34.3%)	1.611 ± 0.555	0.058	0.809	1.749 ± 0.477	1.972	0.161	1.714 ± 0.478	3.016	0.083
Positive	140 (65.7%)	1.591 ± 0.546			1.845 ± 0.468			1.862 ± 0.638		
**EBV infection**										
Negative	178 (83.6%)	1.583 ± 0.559	0.835	0.361	1.808 ± 0.474	0.069	0.793	1.786 ± 0594	1.934	0.165
Positive	35 (16.4%)	1.676 ± 0.486			1.831 ± 0.469			1.938 ± 0.567		

^a^CagA virulence factor was detected in patients with *H. pylori* infection. SD, Standard Deviation; EBV, Epstein-Barr virus; TNM, The TNM Staging System is based on the tumor (T), the extent of spread to the lymph nodes (N), and the presence of metastasis (M). ^**^ *P* < 0.01; ^****^ *P* < 0.0001. ^*^Data are expressed as mean ± standard deviation (SD) of fold change in gene expression level in the gastric tumors normalized to the *ACTB* gene and relative to levels in the adjacent non-neoplastic control sample.

**Figure 3:**
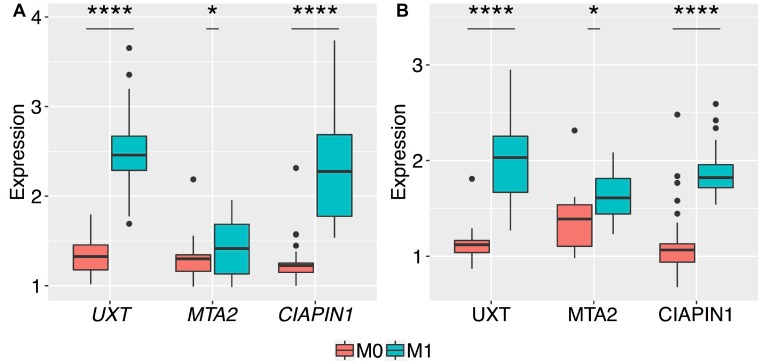
Box plots of the normalized relative expression of *UXT*, *MTA2*, and *CIAPIN1* genes in the gastric tumor tissues of patients without metastasis (M0) and with metastasis (M1). The expression levels of the 3 genes were validated by qRT-PCR and western blot in 68 patients, presented with no serosal invasion (T1 and T2). A highly significant increase in the expression of (**A**) mRNA, and (**B**) proteins of *UXT* and *CIAPIN1* genes between M0 and M1 stages (^****^ *P* < 0.0001). The *t*-test produced a higher *P*-value for differences in the mean expression of mRNA and protein of *MTA2* as compared to those of other genes (^*^ *P* = 0.03 and ^*^ *P* = 0.01, respectively). The boxes are drawn from the 75th percentile to the 25th percentile. The horizontal line inside the box represents the median. Vertical lines above and below the box delineate the maximum and minimum values, and the dots show the outliers.

In order to evaluate *CIAPIN1*, *MTA2*, and *UXT* genes for their potential role in predicting distant metastasis in patients with early-stage GC, we compared their mRNA and protein expression profiles in M0 and M1 patients, including only patients with non-invasive GC (T1/T2) and lymph node negative patients (N0) (*n* = 68). In this study, no patient was at both N0 and M1 at the same time. Thus, only patients with T1/T2 primary tumor could be effectively compared. The results are summarized in Figure 3. The mRNA and protein levels of *MTA2* were significantly different (^*^ *P* < 0.05) between early-stage GC tissues of M0 and M1 patients; however, these differences were more prominent for *UXT* and *CIAPIN1* genes (^****^ *P* < 0.0001). Thus, only *UXT* and *CIAPIN1* genes were considered for further analysis.

**Figure 4:**
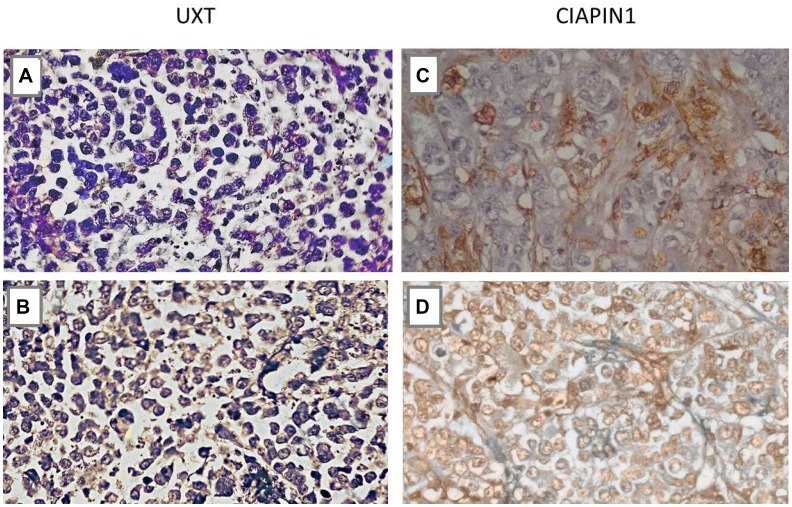
Immunohistochemistry analysis of the UXT and CIAPIN1 proteins in primary gastric tumor tissues of patients without metastasis (M0) and with metastasis (M1). (**A**) Negative sample for UXT (M0), (**B**) Cytoplasmic expression of UXT (M1), (**C**) Negative sample for CIAPIN1 (M0), (**D**) Nuclear expression of CIAPIN1 (M1). More than 10% of cells were stained positively (^*^ *P* < 0.05) (×400 magnification).

Consistent with the qRT-PCR and western blot results, the immunoreactivity of the anti-UXT and anti-CIAPIN1 antibodies showed statistically significant differences between gastric tissue samples from M0 and M1 patients (Figure 4).

### Survival analysis

**Figure 5:**
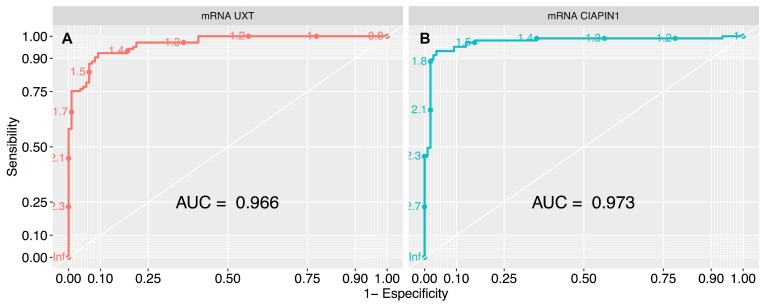
ROC curve analysis to define the cut-off values of *UXT* and *CIAPIN1* gene expression, segregating the high and low expression groups. The largest total area under the curve (AUC) is 0.966 for (**A**) *UXT* gene (that represents normalized expression cut-off of 1.5), and 0.973 for (**B**) *CIAPIN1* gene (cut-off of 1.7). ROC, receiver-operating characteristic.

**Figure 6:**
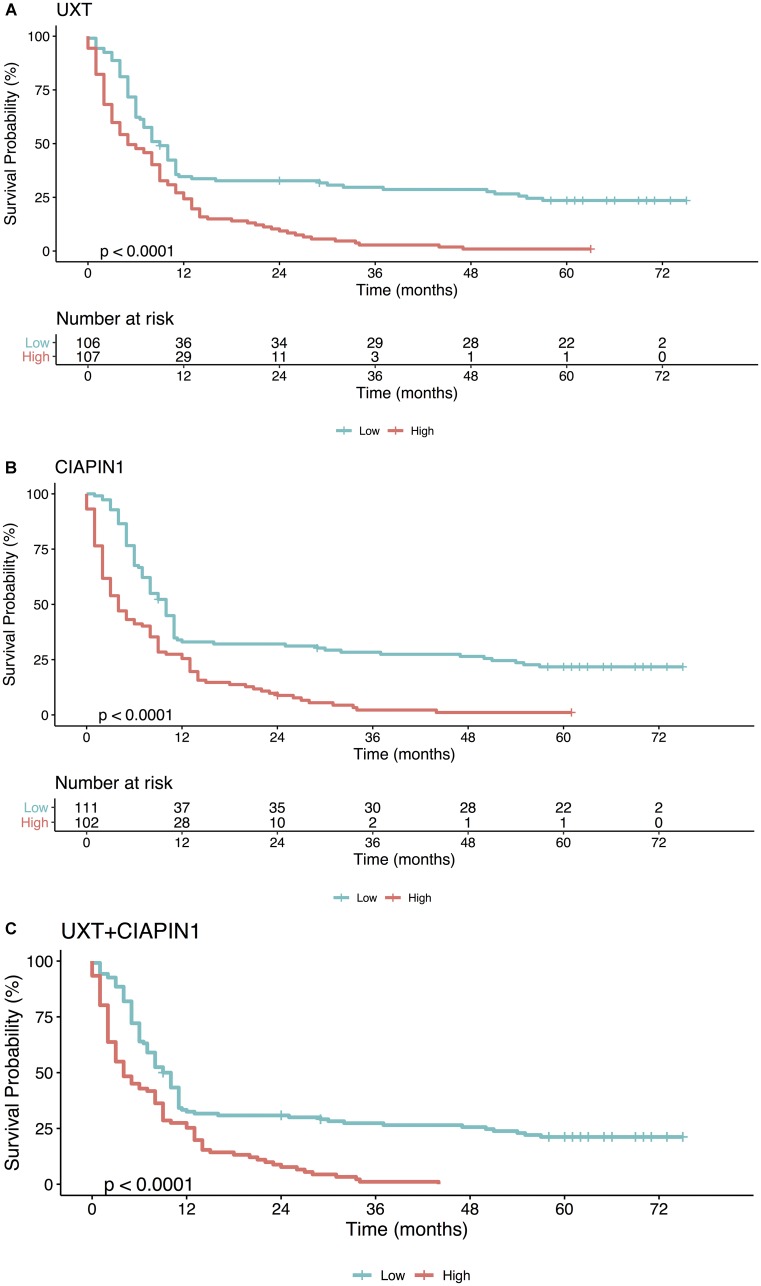
Kaplan–Meier analysis of the overall survival (in months) of patients with gastric cancer as a function of (**A**) *UXT*, (**B**) *CIAPIN1,* and (**C**) gene *UXT* + *CIAPIN1* expression. High expression (gene expression ≥1.5 for *UXT* and ≥1.7 for *CIAPIN1*; red line), as opposed to low expression (gene expression <1.5 for *UXT* and <1.7 for *CIAPIN1*; blue line), is strongly associated with a lower probability of survival (^****^ *P* < 0.0001).

We used survival analysis to evaluate the contribution of high expression of *UXT* and *CIAPIN1* in the overall survival of 213 patients studied. Initially, we used ROC curve analysis to classify the patients into high and low expression groups (Figure 5). The cut-off was chosen as the highest AUC point (for *UXT*: AUC = 0.966; sensitivity = 92.3%, specificity = 90.7%, and for *CIAPIN1*: AUC = 0.973; sensitivity = 93.3%, specificity = 96.2%). From this data, cut-off values were fixed as 1.5 for *UXT* and 1.7 for *CIAPIN1*. Kaplan–Meier analysis demonstrated a decrease in the probability of survival in the group of patients with high expression of *UXT* or *CIAPIN1* (considering individual effects as well as combined effects). In the first year post-diagnosis, there was a highly significant decrease in the overall survival of patients with an increased expression of *UXT* and/or *CIAPIN1* (^****^ *P* < 0.0001) (Figure 6).

## DISCUSSION

A variety of distinct molecular mechanisms may converge in gastric carcinogenesis that is collectively attributed to a combination of environmental factors, and generalized and specific genetic and epigenetic alterations [2]. *MYC* amplification/overexpression is a recurring molecular event in gastric tumor tissues, and is often associated with an advanced tumor stage, lymph node metastasis, and low survival rates [5–7, 10–12]. The *MYC* proto-oncogene coordinates a plethora of cell functions, from cell growth to apoptosis and metabolism. Its deregulation is associated with the global reprogramming of gene expression that promotes cancer initiation, survival, growth, and metastasis; thus considered as a driver event in GC [8, 13]. However, the functional role of MYC in regulating the global expression of genes that participate in critical molecular pathways governing the GC progression is still not well understood [14].

Clinical outcomes of patients with GC depend on the metastatic potential of the tumor. Thus, several studies have focused on detecting prognostic biomarkers capable of predicting metastasis and identifying high-risk patients for guiding treatment decisions [15–17]. In this study, we obtained a whole transcriptome profile of a metastatic GC cell line upon *MYC* silencing, and on comparing with the non-silenced control, we identified at least 150 genes upregulated by MYC that can be explored for GC prognosis. We selected 3 genes for further validation in clinical tumor samples of 213 GC patients. Our qRT-PCR and western blot results revealed a robust increase (^****^ *P* < 0.0001) in *CIAPIN1*, *MTA2*, and *UXT* gene expression in GC tissues of patients that were positive for serosal invasion, lymph node metastasis, and distant metastases, when compared with paired normal gastric tissues. These results suggested that the expression of selected genes had a strong association with advanced-stage GC and, consequently, worse prognosis. We also demonstrated that *UXT* and *CIAPIN1* overexpression was detectable in early-stages of gastric primary tumors, thus enabling the evaluation of possible metastatic progression. However, high expression of *MTA2* seemed to be an event occurring later in the GC evolution.

MTA2 is a member of the metastatic tumor-associated family of transcriptional regulators, and plays a central role in cytoskeletal organization and motility pathways, which are essential processes in the metastatic cascade [18]. As observed in this study, Zhou et al. also showed that *MTA2* expression was closely related to the depth of tumor invasion, lymph nodes metastasis, and TNM staging in patients with GC [19]. In their study, *MTA2* knockdown impaired invasion and metastasis of GC cells. However, another study demonstrated that *MTA2* overexpression enhanced colony formation and tumor growth of GC cells, but was not important in cancer cell migration and metastasis [20]. Such data may suggest that MTA2 is essential for metastasis maintenance and expansion rather than for its initiation. In our study, *MTA2* overexpression was significantly more pronounced in tumor tissues of patients ≥ 61 years of age, and in those with intestinal GC, a histologic type associated with better prognosis as compared to diffuse GC [3]. These findings led us to believe that *MTA2* expression has low specificity as a biomarker of tumor progression and prognosis of GC.

In contrast, this study supported the *CIAPIN1* and *UXT* gene expression analysis as an important strategy to evaluate the probability of occurrence of distant metastases in patients with early-stage GC. Consistently, our Kaplan–Meier survival analysis demonstrated that the survival time of patients with GC with high expression of *CIAPIN1* and UXT (individual or combined) was shorter than those with low expression, during the first year after the diagnosis (^****^ *P* < 0.0001). This result reinforces the prognosis value of these genes for GC, with high sensitivity (92.3% for *UXT* and 93.3% for *CIAPIN1*), and specificity (90.7% for *UXT* and 96.2% for *CIAPIN1*) in the ROC curve.

CIAPIN1 is a ubiquitously expressed protein in differentiated and metabolically active tissues, and exhibits antiapoptotic activity. In a murine cell line, CIAPIN1 was shown to be a downstream effector of the receptor tyrosine kinase belonging to Ras signaling pathway [21]. The role of CIAPIN1 in cancer progression and metastasis is not yet defined. Consistent with our results, higher *CIAPIN1* mRNA expression was associated with poor overall survival in patients with metastatic ovarian serous carcinoma [22]. However, decreased expression of *CIAPIN1* correlated with poor prognosis in patients with other types of cancers, such as colorectal cancer and esophageal squamous cell carcinoma [21–23].

In the SGC7901 and MKN45 GC cell lines, it was shown that reduction in *CIAPIN1* expression promoted tumor growth, suggesting that CIAPIN1 may act as a suppressor of GC cell proliferation [24]. However, both cell lines, although established from metastatic gastric adenocarcinoma [25, 26], do not harbor *MYC* gene amplification [27], which is in contrast to the AGP01 cell line used in the present study. In another study carried out in GC cells, *CIAPIN1* silencing inhibited cell proliferation and angiogenesis [28], suggesting that this gene is important for initiation of tumor vascularization. MYC plays a central role in the recruitment of angiogenic proteins, especially in rapidly proliferating tumor tissues, with high levels of hypoxia [29]. Therefore, a possible explanation for the results obtained in the present study is that CIAPIN1 may be recruited by MYC to maintain the angiogenesis required for tumor progression. A recent study has shown that CIAPIN1 is involved in the inhibition of hypoxia-induced apoptosis, in cardiomyocytes [30]. CIAPIN1 overexpression has also been shown to confer resistance to cancer treatment, therefore predicting a worse patient prognosis and survival [31–33].

UXT is a co-chaperone molecule that assists in the proper folding of proteins, and prevention of cell protein aggregation. It is characterized as a centrosomal protein, and abnormality in its function may cause defects in chromosome separation that may result in cell malignant transformation [34]. Although UXT is highly expressed in several types of human cancers [34, 35], the exact mechanism of its contribution to tumorigenesis and cancer progression is still not understood, and seems to be tissue specific [36]. An important aspect is that UXT can interact physically and functionally with transcription factors, and can act as tumor suppressor or oncogene [36, 37].

Detailed studies of UXT in GC are very scarce. Our report suggested that *UXT* is a *MYC*-regulated gene that is highly expressed in GC tissues in early or advanced stages of tumor progression. Consistently, a study suggested *UXT* as one of the *MYC*-regulated genes, which were highly predictive of poor prognosis in diverse MYC-associated malignancies of epithelial, hematopoietic, or neuroectodermal origin [38].

GC is a highly heterogeneous disease, and classification systems are useful in identifying tumor subtypes with different behavioral patterns (such as aggressiveness, chemotherapy sensitivity, and prognosis) to personalize treatment. Classically recognized systems are based primarily on histopathological and clinical differences. More recently, molecular classification criteria have emerged as an alternative capable of better translating the clinical heterogeneity of GC. The Cancer Genome Atlas (TCGA) project, for example, identified the following four molecular subtypes of GC based on clinical findings and genomic changes: chromosomal instability (CIN), microsatellite instability-high (MSI), genomically stable (GS), and Epstein-Barr virus (EBV) [39]. Our study did not gather enough details to assign molecular subtypes to the tumors of GC patients. More comprehensive studies, similar to the one conducted by Cristescu et al. [40], may reveal whether the gene expression patterns of CIAPIN1 and UXT (as well as the other genes listed in the Supplementary Table 1) may serve as effective biomarkers of one or more molecular subtypes.

In conclusion, our results suggest that the expression analysis of *CIAPIN1* and *UXT* may predict metastasis and poor prognosis in patients with GC. Furthermore, this study revealed a large panel of genes, whose overexpression is directly or indirectly associated with *MYC* amplification. These genes can be further explored as strong candidates for biomarkers of GC prognosis and even for the early diagnosis.

## MATERIALS AND METHODS

### *MYC*-silencing in AGP01 cells

This study was initially conducted to silence *MYC* gene expression in a metastatic GC cell line (AGP01) overexpressing this gene, as previously described [41]. Briefly, a total of 1 × 10^5^ cells were seeded into 12-well cell culture plates, and *MYC* silencing was done by transfecting AGP01 cells with 3 different small interfering RNAs (Silencer Select siRNAs: *s9129*, *s9130*, and *s9131*; Thermo Fisher Scientific, USA). In parallel, AGP01 cells designated “non-silenced AGP01 cells” were transfected with nontargeting siRNAs to serve as a negative control. Silencer^®^ siRNA Starter kit (Ambion, USA) was used for all siRNA experiments. Relative *MYC* mRNA levels were measured by real-time quantitative PCR (qRT-PCR assay ID: Hs00153408_m1; Thermo Fisher Scientific), and MYC protein levels were quantitated by western blot. As previously demonstrated, when the AGP01 cell line was treated with siRNA, *MYC* mRNA and protein expression were reduced more than 70% compared to non-silenced cells. Cell invasion and migration assays, as well as MTT assay indicated that the cells remained viable after transfection [41]. All experiments were performed in triplicate.

### RNA/protein extraction and cDNA synthesis

The extraction of total RNA and proteins from AGP01 cell line, with or without siRNA-mediated silencing of *MYC* gene was performed using the AllPrep DNA/RNA/Protein Mini kit (Qiagen, USA), according to the manufacturer’s instructions. Qualitative and quantitative analysis of RNA and proteins was performed using NanoDrop 2000c spectrophotometer (Thermo Fisher Scientific). RNA integrity was determined by the RNA integrity number (RIN), using Bioanalyzer 2100 platform (Agilent Technologies, USA) [42]. Total RNA was reverse transcribed to cDNA with High Capacity cDNA Reverse Transcription Kit (Applied Biosystems, USA), according to the manufacturer’s instructions.

### RNA-seq

AGP01 cell line transcriptome obtained before and after *MYC* silencing are available in the Gene Expression Omnibus (GEO) database (access codes GSM2147866 and GSM2147867, respectively). RNA-seq was performed using Ion Proton™ platform (Thermo Fisher Scientific). Detailed methods have been described previously [43]. Crude readings were subjected to quality control check, after which the clean readings were aligned to human genome reference sequences (Hg19/GRCh37). The aligned reads were mapped and quantified using TMAP (Torrent Mapping Alignment Program), which supports different alignment algorithms [44–46]. The selection of DEGs was done using bioinformatics and statistical tools, including levels of gene expression and deep analysis. Among the 150 most downregulated genes in the *MYC*-silenced samples, 3 randomly selected genes (*CIAPIN1*, *MTA2*, and *UXT*) were evaluated by qRT-PCR, western blot, and immunohistochemistry of tumor tissue samples from patients with GC.

### Patients

To validate the prognostic relevance and predictive effects of the selected DEGs, clinicopathological data and tumor samples were obtained from 213 patients with GC admitted at João de Barros Barreto University Hospital (HUJBB) in Belém, state of Pará, Brazil, between January 2004 and May 2018. Clinical data and tissue samples (including paired non-neoplastic tissues) of patients treated without preoperative chemotherapy were obtained along with informed consent, with approval from the Ethics Committee of the HUJBB. Tissues were frozen in liquid nitrogen, and stored at –80° C prior to RNA and protein isolation. All tumor samples were histologically diagnosed as gastric adenocarcinoma, and were categorized according to the gender and age variables, tumor location, *H. pylori* infection (by rapid urease test, urea breath test, histological examination, and CagA virulence factor evaluation by PCR), Epstein-Barr virus infection (by *in situ* hybridization test), Lauren’s histological classification [47], tumor length (T), lymph node metastasis (N), presence of distant metastasis (T), and staging, according to the TNM system of the American Joint Council on Cancer (AJCC) [48].

The extraction of total RNA, total proteins, and cDNA synthesis was performed same as the methodology described for AGP01 cell line, and RNA integrity was evaluated by GelRed-stained (Biotium, USA) agarose gel electrophoresis.

### qRT-PCR

The qRT-PCR assay was performed using 96-well optical plates at following conditions: 95° C for 10 min, followed by 40 cycles of 95° C for 15 sec, and 60° C for 1 min, in a StepOnePlus Real-Time PCR System (Applied Biosystems), according to the TaqMan Gene Expression Master Mix (Applied Biosystems) protocol. The threshold cycle (C_T_) values were determined as per default instrument settings. Relative quantification of gene expression was performed according to Livak and Schmittgen method [49], using *ACTB* as reference gene (4333762F; Thermo Fisher Scientific). A corresponding non-neoplastic control sample was used as a calibrator for each tumor sample. Data are expressed as mean ± standard deviation (SD) of fold change in gene expression level in the gastric tumors normalized to the *ACTB* gene and relative to levels in the adjacent non-neoplastic control sample.

### Western blot

Western blot analysis was performed as described previously by Tu *et al*. [50]. Briefly, primary cancer samples were processed, applied on SDS-polyacrylamide gel, and subjected to electrophoresis. Next, individual proteins in the electrophoresis gel were transferred to a polyvinylidene fluoride membrane and labeled with antibodies specific for the selected DEGs proteins. Immunocomplexes were detected by the chemiluminescence method using the ECL Advance Western blotting kit (GE Healthcare Lifesciences).

### Immunohistochemistry

The immunoreactivity of the proteins encoded by selected genes was assessed by immunohistochemistry (anti-CIAPIN1 #PA529259, and anti-UXT #PA518852; Thermo Fisher Scientific) on paraffin-embedded tissue sections. The streptavidin/biotin-peroxidase method described by Hsu *et al*. was adopted [51], using the modifications suggested by Calcagno *et al*. [10]. The normality parameter was defined using normal (non-tumoral) gastric tissue samples fixed in formamide and included in paraffin, obtained from the routine material. Immunoreactivity detected in more than 10% of the histological section cells was considered as positive expression.

### Statistical analysis

The methods and parameters used to identify DEGs from *MYC* silenced and non-silenced AGP01 cells have been previously described [43]. Results of qRT-PCR and western blot were analyzed in QuantStudio Flex Real-Time PCR Software (version 1.2.2, Thermo Fisher Scientific). The unpaired Student’s *t*-test was selected to evaluate the statistically significant differences.

ANOVA followed by Tukey’s test were used to evaluate the association of selected gene expression pattern with clinicopathological variables. Gene expression levels were divided into ‘high’ and ‘low’ using the receiver maximum likelihood (ROC) and area under the ROC curve (AUC). The highest AUC identified the best cut-off expression level that was considered for the Kaplan–Meier analysis. Patients who did not have the event (death) were censored on the last follow-up date or after 6 years and 3 months. The Kaplan–Meier log-rank test was used to estimate the probability of survival for the groups below or above the cut-off for each selected gene. In the statistical analysis (GraphPad Prism 6.0 and R 3.0.2), values of ^*^ *P*< 0.05, ^**^ *P* < 0.01, ^***^ *P* < 0.001, and ^****^ *P* < 0.0001 were considered statistically significant.

## SUPPLEMENTARY MATERIALS





## Abbreviations

GC: gastric cancer
RNA-seq: RNA sequencing
siRNA: small interfering RNA
DEGs: differentially expressed genes
qRT-PCR: real-time quantitative PCR
ROC: receiver maximum likelihood
AUC: area under the ROC curve
MTT: 3-(4, 5-dimethylthiazol-2-yl)-2, 5 diphenyl tetrazolium bromide
ID: identification
GEO: Gene Expression Omnibus
TMAP: Torrent Mapping Alignment Program
HUJBB: João de Barros Barreto University Hospital
ANOVA: analysis of variance
FDR: false discovery rate
