# Supplementary material for: New prognostic markers revealed by RNA-Seq transcriptome analysis after MYC silencing in a metastatic gastric cancer cell line
Source: Oncotarget. 2019 Oct 8;10(56):5768–79. doi: 10.18632/oncotarget.27208 (PMC6791377; doi:10.18632/oncotarget.27208)
Supplement: Supplementary file 1 [file oncotarget-10-5768-s001.pdf]

New prognostic markers revealed by RNA-Seq transcriptome analysis after *MYC* silencing in a metastatic gastric cancer cell line

SUPPLEMENTARY MATERIALS

Supplementary Table 1: The top 150 DEGs downregulated for the combined dataset of MYC silenced versus non silenced AGP01 cells. See Supplementary Table 1
